# Functional genomics to identify the factors contributing to successful persistence and global spread of an antibiotic resistance plasmid

**DOI:** 10.1186/1471-2180-14-168

**Published:** 2014-06-24

**Authors:** Jennifer L Cottell, Howard TH Saw, Mark A Webber, Laura JV Piddock

**Affiliations:** 1 Institute of Microbiology and Infection, School of Immunity and Infection, The College of Medical and Dental Sciences, The University of Birmingham, Birmingham B15 2TT, UK

**Keywords:** Beta-lactam, ESBL, Mobile genetic element, Plasmid, Recombination

## Abstract

**Background:**

The spread of bacterial plasmids is an increasing global problem contributing to the widespread dissemination of antibiotic resistance genes including β-lactamases. Our understanding of the details of the biological mechanisms by which these natural plasmids are able to persist in bacterial populations and are able to establish themselves in new hosts via conjugative transfer is very poor. We recently identified and sequenced a globally successful plasmid, pCT, conferring β-lactam resistance.

**Results:**

Here, we investigated six plasmid encoded factors (*tra* and *pil* loci; *rci* shufflon recombinase, a putative sigma factor, a putative *parB* partitioning gene and a *pndACB* toxin-antitoxin system) hypothesised to contribute to the ‘evolutionary success’ of plasmid pCT. Using a functional genomics approach, the role of these loci was investigated by systematically inactivating each region and examining the impact on plasmid persistence, conjugation and bacterial host biology. While the *tra* locus was found to be essential for all pCT conjugative transfer, the second conjugation (*pil*) locus was found to increase conjugation frequencies in liquid media to particular bacterial host recipients (determined in part by the *rci* shufflon recombinase). Inactivation of the pCT *pndACB* system and *parB* did not reduce the stability of this plasmid.

**Conclusions:**

Our findings suggest the success of pCT may be due to a combination of factors including plasmid stability within a range of bacterial hosts, a lack of a fitness burden and efficient transfer rates to new bacterial hosts rather than the presence of a particular gene or phenotype transferred to the host. The methodology used in our study could be applied to other ‘successful’ globally distributed plasmids to discover the role of currently unknown plasmid backbone genes or to investigate other factors which allow these elements to persist and spread.

## Background

Plasmids have been indispensable tools in the development of molecular biology and much of our understanding of their biology has been based on a small number of model replicon transmissible elements. However, less is known about natural plasmids and in particular, the interplay between plasmids and their host strains. Bacterial plasmids are widely recognised for their role in the expansion and dissemination of virulence and antibiotic resistance genes both between members of the same species and to new bacterial hosts of different species [1,2]. Their ability to acquire and spread either single or multiple antibiotic resistance genes to pathogens has become a considerable problem and an obstacle to successful therapeutic treatment [3]. This is compounded by the lack of development of new effective antibiotics, particularly against infections caused by Gram negative bacteria with plasmid mediated antibiotic resistances, which are causing significant global clinical problems [4]. The recent emergence of genes including β-lactamases which confer resistance to the commonly used β*-*lactam class of antibiotics, can largely be attributed to the spread and persistence of successful plasmids in a wide range of bacterial hosts [5-7]. However, despite their importance and the recently generated wealth of plasmid sequence data [8], our knowledge of the factors which allow plasmids to maintain antibiotic resistance genes, to remain stable in bacterial populations in the absence of selective pressure, and to successfully spread to different bacterial strains is very poor.

In elementary terms the evolutionary success of a plasmid is reliant on (1) the ability to transfer vertically to daughter cells of the host bacterial strain, therefore remaining stable within this population; and/or (2) the ability to transfer horizontally to alternative bacterial hosts via conjugation [9]. Vertical stability can be ensured by the presence of an addiction system such as toxin-antitoxin systems [10]; by lack of a fitness cost conferred by the plasmid [11]; by action of an active plasmid partitioning system [12]; and/or by providing beneficial attributes such as antibiotic resistance or adhesive properties to the host providing a competitive advantage [13]. Effective horizontal transmission is associated with the frequency with which a plasmid can pass between strains and become established in a host strain after conjugation under different environmental conditions [14].

Previously, we sequenced and characterised an IncK plasmid, denoted pCT, isolated from scouring calves [15-17]. Although it was initially identified in *E. coli* animal isolate, the ca. 94 kb plasmid carrying a single antibiotic resistance gene (*bla*_CTX-M-14_) was shown to have disseminated worldwide in bacteria from humans and animals [15] and to stably persist in the host population in the absence of antibiotic pressure [15,18]. Inactivation of the antibiotic resistance gene (*bla*_CTX-M-14_) on pCT also had no effect on the plasmid or bacterial host biology in the absence of selective antibiotic pressure [18]. Therefore, we proposed that alternative plasmid encoded factors were responsible for the successful persistence and global distribution of pCT. In order to test this hypothesis, we used an inactivation technique adapted from a novel gene inactivation method previously used on multi-copy plasmids [18,19] to systematically inactivate candidate genes and operons previously associated with ‘plasmid success’. Using a functional genomic approach analogous to that which has been broadly employed in studying chromosomal genes of various eukaryotic and prokaryotic organisms, we examined the impact of plasmid genes on pCT persistence and conjugation and upon the bacterial host.

## Results and discussion

### Inactivation of six selected genes

**Figure 1:**
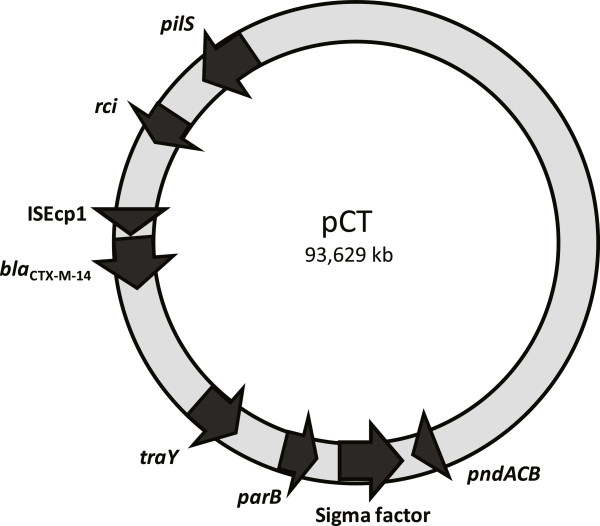
Plasmid map of pCT showing the relative positions of each target genes.

Based upon our previous work [15,18], six loci on pCT were identified as candidates predicted to encode fundamental factors contributing to the success of this plasmid. Comparative genomics with other characterised Incompatibility group I plasmids (including IncI, IncB, IncK and IncZ) identified: a region of pCT encoding a toxin-antitoxin addiction system, *pndACB* (pCT_065) which we hypothesised to be involved in stable inheritance of the plasmid into daughter cells [20]; operons involved in plasmid conjugation, the *tra* and *pil* loci (pCT_068 and pCT_103) [21] including a gene likely to determine mating pair recipient specificity, shufflon recombinase gene *rci* (pCT_093) [22]; an unusual putative sigma 70 factor (pCT_066) and a putative *parB* gene involved in plasmid segregation (pCT_057) [15]. Therefore, the effects of inactivating the *pndACB* operon*, rci,* pCT_066 and key structural pilus protein genes *traY* (*tra* locus)*, pilS* (*pil* locus) and the putative *parB* gene were investigated to establish the role of each element in plasmid ‘success’ (Figure 1).

Each gene was inactivated by homologous recombination using hybrid amplimers encoding an *aph* cassette encoding kanamycin resistance, flanked by regions homologous to the target. Mutants were created within an intermediate Lambda Red recombinase encoding *E. coli* SW102 host [23] and confirmed by sequencing across the mutated region to ensure the *aph* cassette has been inserted to inactivate the target gene. All six recombinant plasmids were then transformed into *E. coli* DH5α, and transferred to *S.* Typhimurium SL1344 to prevent further recombination events and for further analysis.

### Inactivation of the six genes had no effect on pCT maintenance

Both wild-type pCT and each of the recombinant pCT plasmids remained stable over the investigated time period (approx. 80 generations) in 100% of both *E. coli* DH5α and *S*. Typhimurium SL1344 host cells (Table 1). These data indicate that none of the six selected pCT genes are individually responsible for the short term maintenance and successful vertical transfer of this plasmid, as their inactivation did not impact on the inheritance of pCT. The *pndACB* operon is homologous to known and characterised systems in other plasmids, such as R64, R483, p026-vir, ColIb-P9 and pO113, with protein identity between 91% and 100%. Furuya and Komano (1996) showed that when the *pndACB* operon, similar to that found on the IncI plasmid R64 was inactivated, R64 was rapidly lost from the bacterial population, therefore it was required for maintenance of R64 over a similar time period [24]. Based on protein homology, plasmid pCT was found to encode a putative *parB-*like nuclease gene which shares 100% identity to a previously characterised ParB protein in p026-vir. However, the putative *parB* gene on pCT shares no significant homology to the *parB* DNA sequences from other IncI plasmids, such as R64 and CoIIb-P9. We found that the recombinant pCT plasmid carrying the inactivated putative *parB* gene also showed no significant difference in stability when compared to the wild-type plasmid. This was in contrast to work by others with plasmid P1, which showed that an intact *parB* is essential for the stable partitioning of P1 [25]. Our data with pCT indicated that neither *pndACB* nor the putative *parB* genes are individually essential for pCT stability under conditions tested suggesting they may not be expressed under such conditions; may work in conjunction with other elements; or are non-essential for stability due to the presence of other currently unidentified genes or gene regions. These data also suggest that broad conclusions about gene function cannot be extrapolated from data obtained with other plasmids.

**Table 1:** Comparison of recombinant plasmids with wildtype pCT plasmid

**Gene inactivated on pCT**	**Stability**	**Conjugation to an *E. coli *** **recipient**	**Conjugation to a *Salmonella *** **recipient**	**Bacterial host growth kinetics**	**Biofilm formation**	**Competitive index when co-cultured with WT pCT**
Sigma factor::*aph*	=	=	=	=	=	1.00
*pilS*::*aph*	=	**↓**	**↓**	=	=	1.00
*traY*::*aph*	=	UD	UD	=	=	0.99
*rci*::*aph*	=	=	**↓**	=	=	0.99
*pndACB*::*aph*	=	=	=	=	=	1.00
*parB::aph*	=	ND	ND	=	=	ND

=, the same as wild-type (WT) pCT; **↓**, reduced rate when compared to pCT; ND, not determined; UD, Undetectable.

### The relative contribution of each conjugation pilus in pCT horizontal transfer

**Figure 2:**
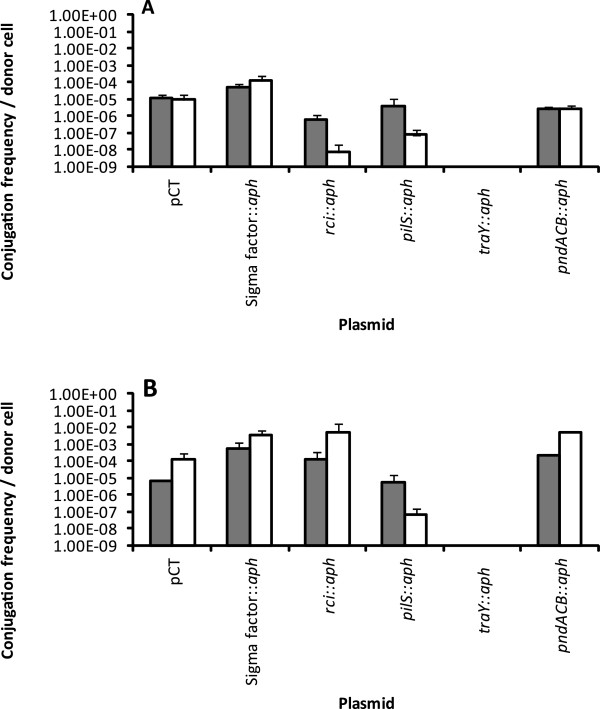
**Conjugation frequencies of wild-type pCT and the pCT mutants on a solid surface (filled box) and in liquid (open box) from bacterial donor *E. coli *** **DH5α to A) a *S. *** **Typhimurium recipient and B) an *E. coli *** **recipient.**

To investigate the contribution of the two conjugation pilus genes (*tra* and *pil*) in the dissemination of pCT, the effects of inactivating the major structural protein genes of each pilus (*traY* and *pilS*) were assessed. Inactivation of *traY* prevented pCT transfer both in liquid and on solid surfaces (Figure 2) confirming the essential role of the *tra* locus for pCT conjugation under both conditions [26]. The inactivation of thin pilus (*pil)* gene, *pilS*, had no effect on the conjugation rate on solid surfaces, but reduced the frequency of pCT conjugation in liquid to both *E. coli* and *S.* Typhimurium recipients (Figure 2). This was in agreement with previous studies which have shown that the *pil* locus is required for conjugation in liquid [21,27]. Removal of an *rci* recombinase, which allows the recombination of shufflon elements to determine the terminal thin pilus protein and impacts on host specificity, has previously been shown to fix this region into one particular conformation [22]. Inactivation of the pCT *rci* gene resulted in a reduced transfer rate of pCT to the *S.* Typhimurium recipient, particularly in liquid media, however there was no effect on the rate of transfer to the *E. coli* recipient (Figure 2). Therefore, we conclude that the thin pilus is not essential for pCT conjugation. However, the presence of the thin pilus consistently increased the frequency with which pCT conjugated into recipient host strains within liquid. It may be that production of the thin pilus provides better attachment of the mating pair in liquid, and the active shufflon region allows variation and an extended pCT bacteria host range as shown in R64 [24]. As inactivation of *pilS* had no effect on pCT transfer on a filter to *E. coli* recipients, the role of the thin pilus in conjugation on a solid surface is less clear (Figure 2, Table 1).

### Inactivation of pCT genes had no detected effect on various bacterial hosts

Inactivation of the six selected genes on pCT in each of the recombinant plasmids had no effect on bacterial host growth rates during mid-logarithmic phase or generation time of either host when compared to hosts containing wild-type pCT (Table 1). Apart from the inactivated *parB*, each mutant plasmid also remained in a 1:1 ratio when *E. coli* DH5α cells containing each mutant plasmid were co-cultured in competition with *E. coli* DH5α containing wild-type pCT *in-vitro*. After approximately 80 generations, cells containing each mutant plasmid had a competition index indistinguishable from 1.0 (Table 1) indicating no fitness advantage or disadvantage over host cells containing wild-type pCT. Therefore, inactivation of the five selected pCT gene regions had neither a beneficial or detrimental effect on host growth or on the host’s ability to compete in co-culture, suggesting these genes do not individually contribute or alleviate any significant burden the plasmid may place on the bacterial host cell under conditions tested. In contrast, the recombinant plasmid carrying the inactivated *parB* gene was out-competed by the wild-type pCT plasmid. The reason behind this phenomenon is unclear as the host cells carrying this recombinant plasmid exhibited no detectable growth defect. Similarly, the recombinant plasmid showed no obvious stability problem within the host. However, we do not exclude the possibility that the recombinant plasmid carrying host may be less fit compared to the wild-type plasmid carrying host over a longer duration of competition.

Inactivation of the six loci also had no effect on the ability of host bacterial cells to form a biofilm (Table 1), suggesting that the selected genes do not contribute to the bacterial host’s ability to do so. These data are in contrast to the findings of Dudley *et al.* (2006) who showed that inactivation of *pilS* on the IncK plasmid, pSERB1, reduced the host bacterium’s ability to form a biofilm by up to 50%, strongly suggesting a role in biofilm formation for the pSERB1 thin pilus [13]. It maybe that other plasmid encoded factors allow for the differences in the ability of the host to form a biofilm, or that the effects on biofilm formation are host specific and only seen under particular environmental conditions. Inactivation of the putative sigma factor (pCT_066) had no detectable effect under any of the conditions tested, suggesting no role in plasmid dissemination or modulation of host bacterial fitness. Further investigation, including transcriptomic experiments are required to determine whether this sigma factor can affect the expression of plasmid or host chromosomal genes and whether our assays were not sufficiently sensitive to detect any subtle effects of removing this gene.

## Conclusions

In conclusion, we postulate that the success of this plasmid is due to a combination of subtle factors rather than one particular gene or phenotypic benefit conferred to host strains. These factors include stability within a range of bacterial hosts (due in part to the presence of numerous genes involved in plasmid stability), a lack of a fitness burden conferred to new host strains allowing establishment of the plasmid in new hosts (shown previously) [18], and proficient conjugation allowing dissemination of pCT to a range of bacterial hosts in both liquid and on solid media. Although it is conventional to believe that the prudent use of antibiotic therapy would reduce the spread and dissemination of antibiotic resistance gene harbouring plasmids, our previous data have suggested otherwise [18]. We have also shown the pCT backbone to be robust in its persistence and not reliant on any single loci tested. This means that the reduction in selection pressures will not always reduce the numbers of bacteria carrying such plasmids with antibiotic resistance genes, and re-exposure to antibiotics will likely amplify the numbers of these antibiotic resistant strains. There is still much to learn about the complex nature of plasmid and bacterial host strain interactions with regard to plasmid functions, such as conjugation, stability and the overall evolutionary fitness of plasmids with their host in different conditions. However, we have shown that the functional genomic approaches we used in our study provide an example of how to use plasmid genomic data to explore fundamental and applied biological questions. We have also been able to inactivate specific loci on several other, globally successful plasmids including those carrying the carbapenemases *bla*_KPC_ and *bla*_NDM-1_, illustrating the utility of our approach and its broad applicability to the study of plasmid gene function (manuscripts in preparation). Recent advances in sequencing have identified various ‘successful’ plasmids such as those found associated with the globally disseminated strain *E. coli* ST131 [7] or those carrying other prominent resistance genes such as *bla*_CMY-2_ or *bla*_NDM-1_. Investigating the factors key to their dissemination could also be examined using a similar approach [28,29]. A better understanding of the biological relevance of plasmid ‘backbone’ genes in the successful survival and spread of antibiotic resistance plasmids will be of paramount importance if we are to prevent future persistence and further spread of both plasmid vectors and the antibiotic resistance genes that they carry.

## Methods

### Bacterial strains and plasmid extraction

Wild-type plasmid pCT [Genbank: FN868832] was isolated from a veterinary *E. coli* strain C159/11 [15,16]. Wild-type pCT and recombinant pCT DNA was extracted using a QIAprep Spin Miniprep Kit (Qiagen, Germany) and a QIAGEN Large Construct Kit (Qiagen, Germany) according to the manufacturers’ instructions. All plasmids were transformed into *E. coli* DH5α electro-competent cells (Bioline, UK) (1.25 kV, 25 μF, 200Ω, in chilled 2 mm electroporation cuvettes) and transformants selected by growing on agar containing 8 mg/L of cefotaxime (Sigma-Aldrich, USA) or 50 mg/L of kanamycin (Sigma-Aldrich, USA) when the *aph* cassette is used for gene inactivation.

### Inactivation of the six selected pCT genes

To inactivate the six selected pCT genes, pCT was transformed into the *E. coli* strain, SW102 which carried a chromosomal Lambda-Red Recombinase [23]. Where transformation of the plasmid into this strain is difficult, conjugation by filter mating was done by selecting the transconjugants on media containing 50 mg/L of tetracycline and 8 mg/L of cefotaxime. The hybrid primers used to inactivate the selected pCT genes were designed to have 20 bp identity to the *aph* cassette on pKD4 [30] and 40 bp sequence identity to the target genes (Table 2). Recombination of amplimers encoding the *aph* gene with each pCT gene was carried out as previously described [18]. Recombination was confirmed in each case by PCR and sequencing across the mutated DNA region (Table 2). The recombinant plasmid was then extracted and electroporated into DH5α or conjugated into another host strain to avoid further recombination from occurring and for further study.

**Table 2:** Primers used in the construction and confirmation of recombinant pCT plasmids

**pCT gene**	**Description**	**Primer sequence**	**Amplimer size (bp) (WT/inactivation)**
Sigma factor pCT_066	Confirmation forward	ACAGCGTCTTCTCGTATCCA	1289/1675
	Confirmation reverse	GTTCTTCCAGCTGACGTAAC	1289/1675
	Recombination 1	GGAGGGCGTCTCGCTAAAAAAACTTACTCAAACACATCAAGTGTAGGCTGGAGCTGCTTC	1574
	Recombination 2	GCATTACTTTTTATTCTCGTGAGACTCAAGGTCATTCGGTGGGAATTAGCCATGGTCCAT	1574
*rci*	Confirmation forward	AAGGTCATCTGCAGGAGT	945/1867
	Confirmation reverse	GTGTCGCAGCAACAATA	945/1867
	Recombination 1	GGGGGACATGCCGTATGAATCCTGTTGAACTGGTCCGAAAGTGTAGGCTGGAGCTGCTTC	1574
	Recombination 2	GCAGTGTCACGACAAACAGCCCGTTTCTGCACCCGACAGTGGGAATTAGCCATGGTCCAT	1574
*pilS*	Confirmation forward	GCGGAAGGAAGTGAGCATAA	722/2053
	Confirmation reverse	CAGTGACATGCTGAAGCAGT	722/2053
	Recombination 1	TGGTGACCAGATCAATACAGTTTTTCTTCGGCACATTGCTGTGTAGGCTGGAGCTGCTTC	1574
	Recombination 2	AACCTGCAGACAATCGCCACCAAAATGAAAGCCCAGAAAGGGAATTAGCCATGGTCCAT	1574
*traY*	Confirmation forward	GGAGAGTCCGGTCTGTATGA	2423/2138
	Confirmation reverse	TGCAACCAGTGTGGTACAG	2423/2138
	Recombination 1	GTATCCTGGTCTGCCTGTTACTGATGAGTACCATTGCAGCGTGTAGGCTGGAGCTGCTTC	1574
	Recombination 2	CGGCACAAAACAGCAAAAACAGCAGGAAGTAGAGTGGTGGGGGAATTAGCCATGGTCCAT	1574
*pndACB*	Confirmation forward	AAGGATTGTGGCGGACAGGA	486/1288
	Confirmation reverse	TGATGACGCACAGGACGGAA	486/1288
	Recombination 1	CCCAGGCGATTTTTTTATCAATCAACCCAGGGCCCACTGTGTGTAGGCTGGAGCTGCTTC	1574
	Recombination 2	ATTGAGGTCAGCCTTCGCAACAATCCGGCGGCAGATGTCCGGGAATTAGCCATGGTCCAT	1574
*parB*	Confirmation forward	TATTAAAAATAACGCGGCGG	663/1872
	Confirmation reverse	GCAAAGTATCACACTGCCAAAA	663/1872
	Recombination 1	GGAGCGGCGGGAGAGTATAGTCATTATTGTAGTCCGGGTAGTGTAGGCTGGAGCTGCTTC	1574
	Recombination 2	CTTTTCACTCACCATTATTTTTTCCGCTTCTCTCTGTGCCGGGAATTAGCCATGGTCCAT	1574

### Conjugation rates

The conjugation rate of recombinant plasmids was measured on a filter (Whatman, USA) placed on an LB agar plate and in LB broth incubated at 37°C with shaking at 180 rpm for three hours as previously described [31]. A rifampicin resistant *E. coli* (DH5α) and *S.* Typhimurium (SL1344) were used as recipient strains and selection of transconjugants on LB agar containing 100 mg/L rifampicin and 8 mg/L cefotaxime (and 50 mg/L of kanamycin). Conjugation frequencies were determined on three separate occasions. Unpaired Student’s *t-*tests were used to determine whether any significant changes were observed in the conjugation frequency (*p <* 0.05). The conjugation rate of the *parB* mutant was not determined due to the confounding effects arising from its instability. This made accurate measurements of plasmid transfer difficult due to an inability to identify host strains which have lost the plasmid.

### Ability to form biofilm

The ability of strains containing each plasmid to form a biofilm was evaluated using crystal violet staining of biofilms formed over 48 hours at 30°C as previously described [32]. Optical density at 600 nm was measured to quantify the amount of biofilm produced on three separate occasions using three biological replicates with four technical replicates each in every experiment. A significant difference was determined by Student’s *t-*test where *p* value was less than 0.05.

### Growth kinetics

Growth of each bacterial strain containing both wild-type pCT and the recombinant plasmids was determined by monitoring the optical density of bacterial cultures at a wavelength of 600 nm in LB broth in a FLUOstar OPTIMA (BMG Labtech, UK) as previously described [33]. The growth kinetics were repeated at least three times with three biological replicates per strain in each experiment and the differences were analysed using unpaired Student’s *t-*test. Differences were significant when *p* value was less than 0.05.

### Plasmid persistence

Stability of the mutant plasmids was measured by assessing the proportion of cells that carry each plasmid over time within LB broth isogenic cultures incubated at 37°C with shaking at 180 rpm. At 12, 24, 48 and 72 hours, 100 μl of culture was used to inoculate fresh pre-warmed LB broth at a dilution of 1:100. Viable counts were determined every two hours for the first 12 hours and then at 24, 48, 72 and 96 hours. Colonies from each viable count were replica plated onto antibiotic free and antibiotic containing agar plates (8 mg/L of cefotaxime or 50 mg/L kanamycin). Colonies growing on the antibiotic free plate but not on the antibiotic containing plates indicated the proportion of bacteria that had lost the plasmid. The experiment was repeated on three separate occasions using three biological replicates of each strain on each occasion.

### Pair-wise competitive growth

A pair-wise competition assay *in-vitro* was used to determine whether inactivation of the six genes on pCT impacted upon the ability of the plasmid to persist when competed within a culture with cells containing wild-type pCT. Overnight bacterial cultures of DH5α pCT and DH5α containing the five pCT mutant plasmids were used to seed fresh LB broth in a 1:1 ratio and grown at 37°C with shaking at 180 rpm. A viable count was performed every two hours and cultures were used to seed fresh broth every 24 hours for a period of 4 days. Colonies from the viable count were replica plated onto LB agar plates containing 1) cefotaxime 8 mg/L, 2) kanamycin 50 mg/L, and 3) no antibiotic. The proportion of each plasmid in each culture was determined at each time point by counting the number of colonies on each of the antibiotic selective plates and calculating the proportion of each test plasmid accordingly. The competition index was defined as 1 + ([log_10_A – log_10_B]/number of generations) modified from Pope *et al.* (2010) [34], where A is the ratio of the plasmids at 72 hours (including four passages), B is the ratio at the beginning of the assay, a competitive index of 1 indicates no competitive advantage nor disadvantage within the assay.

## Competing interests

The authors declare that they have no competing interests.

## Authors’ contributions

JLC and HTHS carried out the experiments and analysed the data. All authors contributed to writing of the manuscript. Experimental strategy was carried out by MAW and LJVP who also supervised the project. All authors read and approved the final manuscript.

## Authors’ information

Jennifer L Cottell and Howard TH Saw: joint first authors.
